# Impacts of incentivizing milk spending among SNAP participants

**DOI:** 10.1093/haschl/qxag193

**Published:** 2026-07-25

**Authors:** Vikas Mishra, Joel Cuffey, Kara Newby, Alicia Powers

**Affiliations:** Hunger Solutions Institute, Auburn University, Auburn, AL 36849, United States; Department of Agricultural Economics and Rural Sociology, Auburn University, Auburn, AL 36849, United States; Department of Agricultural Economics and Rural Sociology, Auburn University, Auburn, AL 36849, United States; Hunger Solutions Institute, Auburn University, Auburn, AL 36849, United States; Hunger Solutions Institute, Auburn University, Auburn, AL 36849, United States

**Keywords:** milk, nutrition incentives, SNAP

## Abstract

**Background:**

Incentive programs to encourage healthier eating among US households generally focus on fruits and vegetables, and little is known about incentivizing other components of a healthy diet. The Healthy Fluid Milk Incentive (HFMI) funds projects that provide discounts for households participating in the Supplemental Nutrition Assistance Program (SNAP) when they purchase lower-fat milk products.

**Methods:**

We used transaction data from a large retailer participating in an HFMI project (Add Milk) to estimate the impact of HFMI discounts on sales of different types of milk. We measured monthly spending among households that consistently purchased milk at the retailer. We used difference-in-differences and event study models to estimate the impact of Add Milk on SNAP households compared with non-SNAP households.

**Results:**

We found that Add Milk discounts increased spending on 1% milk among SNAP households by up to 21.0% and did not impact fat-free milk spending. The program took over 6 months to see an impact. Sales of higher-fat milk were reduced and sales of flavored milk were not affected.

**Conclusion:**

These findings demonstrate the feasibility of incentivizing dietary components beyond fruits and vegetables but the difficulty of inducing substitution away from less-healthy items.

## Introduction

Low-income households are often observed to make food choices that are dense in calories and nutritionally less diverse,^1,2^ which may increase vulnerability to diet-related chronic diseases.^3^ US food and nutrition policy discussions therefore often focus on improving diets of low-income households. One set of policies offers low-income consumers incentives to purchase healthy food, primarily fruits and vegetables.^4^ Such incentive programs have proven effective in increasing fruit and vegetable spending and consumption.^5-7^ In addition to fruits and vegetables, other parts of a healthy diet include whole grains and lower-fat protein sources such as beans and milk.^8^ Much less is known about the impact of incentives for other healthy food products.

The 2018 Farm Bill established the Healthy Fluid Milk Incentive (HFMI) pilot project to incentivize unflavored and unsweetened fat-free (skim) and 1% milk consumption among Supplemental Nutrition Assistance Program (SNAP) participants. HFMI has expanded over multiple years and is currently branded as Add Milk. As of September 2025, Add Milk was offered at participating retailers in 22 states across the United States. Add Milk retailers range from small grocery stores to supermarkets, and most locations are part of a regional grocery chain. Despite the possible role of milk in contributing to child growth^9^ and adult health,^10^ milk consumption has been dropping in the United States over the past few decades.^11^ While there is controversy around whether fat-free and 1% milk is healthier than higher-fat milk,^12^ incentivizing unflavored and unsweetened lower-fat milk may be beneficial if households substitute flavored milk products with unflavored and unsweetened lower-fat milk^13^ or increase milk consumption overall.

The goal of this study was to investigate the impact of the Add Milk program on SNAP household spending on different types of milk. We used information on all milk transactions at a large retailer participating in Add Milk and difference-in-differences (DID) and event study frameworks to estimate the impact of Add Milk on fat-free milk spending, 1% milk spending, whole and 2% milk spending, and flavored milk spending.

## Data and methods

### Study setting

The Add Milk program is supported by the US Department of Agriculture's (USDA’s) Food and Nutrition Services and is currently operated by the Hunger Solutions Institute, Auburn University. Add Milk offers incentives in the form of immediate discounts at the point-of-sale to customers when they use their SNAP Electronic Benefits Transfer (EBT) card to buy qualifying liquid cow-milk products at participating retailers. Incentivized milk was unflavored and unsweetened fat-free and 1% milk for the period of this study, in alignment with the 2020–2025 Dietary Guidelines for Americans.

This study used data from 1 participating retailer operating 261 supermarkets across 6 US states. Add Milk was launched simultaneously in all of this retailer's stores in the third week of December 2023. No other retailer participated in Add Milk in these 6 states during the study period. Initially, the program offered a 20% immediate discount when SNAP households purchased fat-free or 1% milk using SNAP benefits. In the second week of April 2024, the discount was increased to 40%. The discount was available for both in-store and online purchases. The retailer was reimbursed for all discounts provided through the Add Milk program. Add Milk was advertised using in-store banners and refrigerator stickers informing customers that they could receive “40% off fat free and 1% milk with your EBT-SNAP card.” Receipts from purchases that included Add Milk discounts clearly denoted these discounts, and the discounts were also advertised to customers through traditional media and social media.

As an immediate discount at the point-of-sale, Add Milk decreased the price of lower-fat milk relative to higher-fat milk for customers using SNAP to buy milk. Among individuals using SNAP, this difference in price would be expected to induce SNAP customers to substitute lower-fat milk for higher-fat milk or other nonmilk products. Additionally, the discount on lower-fat milk may free up more resources and enable SNAP buyers of lower-fat milk to buy more total milk or nonmilk products. Since our data came from 1 retailer, we did not observe total milk spending among SNAP households. This study therefore focused on measuring the impact of Add Milk on substitution between milk products at this retailer instead of the impact on total milk spending.

In addition to causing regular SNAP customers to substitute towards lower-fat milk and increase total spending, the discount may also bring in new customers or induce SNAP households to pay with SNAP benefits instead of cash. Our data from a single retailer do not allow us to observe milk spending prior to the program among customers who switch to this retailer because of the program. We therefore restricted our analysis to households who regularly shopped at the retailer both before and after the launch of the program.

### Data and sample

We obtained summary information on all transactions in which a customer used a SNAP EBT card as well as all fluid milk transactions at the 261 stores between May 2023 and July 2025. We chose May 2023 as the first analysis month to avoid potential spillover from the end of the SNAP Emergency Allotments in February 2023 in 4 of the 6 states. Information on each transaction included prediscount value of spending on fat-free, 1%, 2%, whole milk, and flavored milk; the number of units sold (of any size) of fat-free, 1%, 2%, whole milk, and flavored milk; and how much was paid using SNAP EBT and other payment types (eg, cash, credit card, debit card).

Each transaction was also linked to a retailer-generated anonymous household identifier. As a regular part of its business operations, the retailer uses checkout information such as payment method and customer loyalty information to identify transactions from a single household. The retailer then generates a household identifier and assigns it to all transactions completed by that household. These household identifiers (and none of the underlying identifying information) were provided to the study team.

We excluded the months of December 2023 and April 2024, when the program either launched or changed its incentive structure, and February 2025 when a technical error resulted in incorrect milk sales reporting. This resulted in summary information on 27 942 672 SNAP transactions (some of which included milk) and 117 743 691 milk transactions (some of which were paid for using SNAP).

We designated a household as a SNAP household if they used a SNAP EBT card to purchase any product (not necessarily milk) every month from May 2023 to July 2025 at this retailer. Non-SNAP households were households that never used a SNAP EBT card at the retailer. To ensure that the composition of SNAP vs non-SNAP households remained the same throughout our study period, we excluded households with SNAP purchases in some months but not others. In addition to mitigating potential bias due to changes over time in the composition of households using SNAP at the retailer, excluding these intermittent SNAP households provided more accurate identification of SNAP vs comparison households. Months in which intermittent SNAP households do not make a SNAP purchase may result from either the household not participating in SNAP—and so being a comparison household—or the household having SNAP but not using it at the retailer.

Using the transaction-level data, we obtained each household's monthly milk spending using any payment method (SNAP or non-SNAP) on fat-free, 1%, whole and 2%, and flavored milk. Spending was measured at shelf prices, prior to discounts being applied. We also obtained each household's monthly number of units purchased (of any size) of fat-free, 1%, whole and 2%, and flavored milk.

As mentioned previously, since we only had data from 1 retailer, to minimize bias from unobserved milk spending at other retailers we restricted the sample to households that regularly shop at this retailer. We defined this sample to be a balanced panel of households that purchased milk every month from May 2023 to July 2025 at this retailer. In separate models, we also considered the robustness of our results to a more restrictive sample of households that consistently purchase substantial amounts of milk at this retailer. We define this more-restrictive sample to include only households that purchase over $2 in milk every month from May 2023 to July 2025 at this retailer.

Our household sample covered milk spending over 24 months by 159 273 non-SNAP households and 4475 SNAP households, providing 3 929 952 household-month observations. Our data consisted of nonidentifiable information already routinely collected by the participating retailer, so the authors' institutional review board determined the study to be not human subjects research.

### Outcomes and covariates

Our main household-level outcomes were the prediscount value of total monthly spending on fat-free milk, 1% milk, whole and 2% milk, and flavored milk. To examine the robustness of these primary outcomes, in separate models we used the total number of units sold of each milk type as the outcome.

Our primary independent variables were indicators for aggregate time periods and an indicator variable for whether the household was a SNAP household. To measure changes between any month after the program's launch relative to prior to the program's launch, we identified all months postlaunch (January 2024–July 2025) using a single indicator variable. To capture dynamic impacts of the program, we also created indicator variables for 1 prelaunch and 3 postlaunch periods. “Pre” spanned 6 months prior to the launch (May 2023–October 2023), “low discount” spanned the 3 months with the 20% discount (January 2024–March 2024, “early high discount” spanned the first 7 months of the 40% discount (May 2024–November 2024), and “late high discount” spanned the last 7 months of the study period with the 40% discount (December 2024–July 2025 excluding February 2025). For additional control variables, we also calculated total milk purchases and total milk transactions over all milk types by each household in each month.

### Statistical analysis

We used DID and event study models to compare trends in spending between SNAP and non-SNAP households. As only SNAP households could receive incentives, non-SNAP households served as the comparison group. This design assumes that milk-spending trends among SNAP households without the program would have followed spending trends among non-SNAP households. While the assumption is untestable, the event study models enabled a comparison of trends prior to program launch and so provided an indication of the assumption's validity.

Independent variables in the DID models included the SNAP household indicator, the indicator for whether the month was after the Add Milk launch, and the interaction between the SNAP household indicator and the indicator for being after the launch. Independent variables in the event study models included indicators for program periods relative to the Add Milk launch (pre, low discount, early high discount, and late high discount), interactions between these indicators and the SNAP household indicator, and household fixed effects. In the event study models, the month just before program launch (November 2023) was the reference period and the parameters of interest were the coefficients on the interactions between period indicators and SNAP.

To ensure that our results isolated changes in substitution between specific milk types from overall changes in total milk spending, both DID and event study models also controlled for total milk spending and the number of milk transactions. In separate models we investigated the robustness of our results to omitting the control for the number of milk transactions. Standard errors for all models were clustered at the household level.

### Limitations

This study used plausible comparison households to estimate the effect of incentivizing select milk but did not experimentally vary which households received incentives. Unobserved factors influencing milk spending may therefore have biased our results. In particular, we did not have information on program awareness or on the timing of retailer marketing efforts about the program. Our data did not contain information about specific nonmilk products purchased, so we were unable to measure the program's impact on other foods or beverages. We also did not observe household characteristics such as income. Finally, we had information from only 1 retailer among several that households may visit and purchase milk from. We were thus unable to capture the impact on total household milk purchases across all retailers, and were unable to measure the impact of the program on households who switched to using SNAP at this retailer from other retailers.

## Results

### Difference-in-differences results

SNAP households increased adjusted spending on 1% milk by $0.12 compared with prior to the Add Milk launch, while non-SNAP households decreased adjusted spending on 1% milk by $0.09 over the same time period (Table 1). Thus, SNAP spending on 1% milk increased by $0.21 (95% CI, $0.15–$0.29) more than non-SNAP spending on 1% milk. There was no statistically significant difference between changes in SNAP and non-SNAP spending on fat-free milk (DID, –$0.02; 95% CI, −$0.10 to $0.07). SNAP household spending on whole and 2% milk decreased by $0.28 more than non-SNAP household spending on whole and 2% milk (95% CI, −$0.39 to −$0.17). Flavored milk spending among SNAP households increased by $0.08 (95% CI, $0.02–$0.14) relative to changes in flavored milk spending by non-SNAP households.

**Table 1:** Adjusted spending of SNAP and non-SNAP households, pre- and post-launch of the Add Milk program.

	SNAP ($)			Non-SNAP ($)			
	Pre	Post	Difference	Pre	Post	Difference	DID (95% CI)
1% Milk	1.67	1.79	0.12	1.75	1.66	−0.09	0.21 (0.15, 0.29)
Fat-free milk	0.70	0.73	0.03	2.06	2.11	0.05	−0.02 (−0.10, 0.07)
Whole and 2% milk	10.19	9.98	−0.21	9.20	9.27	0.07	−0.28 (−0.39, −0.17)
Flavored milk	1.76	1.81	0.05	1.30	1.27	−0.03	0.08 (0.02, 0.14)

Abbreviations: DID, difference in differences; SNAP, Supplemental Nutrition Assistance Program.

Source: Authors' analysis of household-month household spending from participating Add Milk retailer. Averages and differences shown for households that used SNAP at the retailer every month (SNAP) and households that never used SNAP (non-SNAP) prior to and after launch of the Add Milk program. Averages and differences adjusted for total milk spending and total milk transactions.

### Event study results

**Figure 1:**
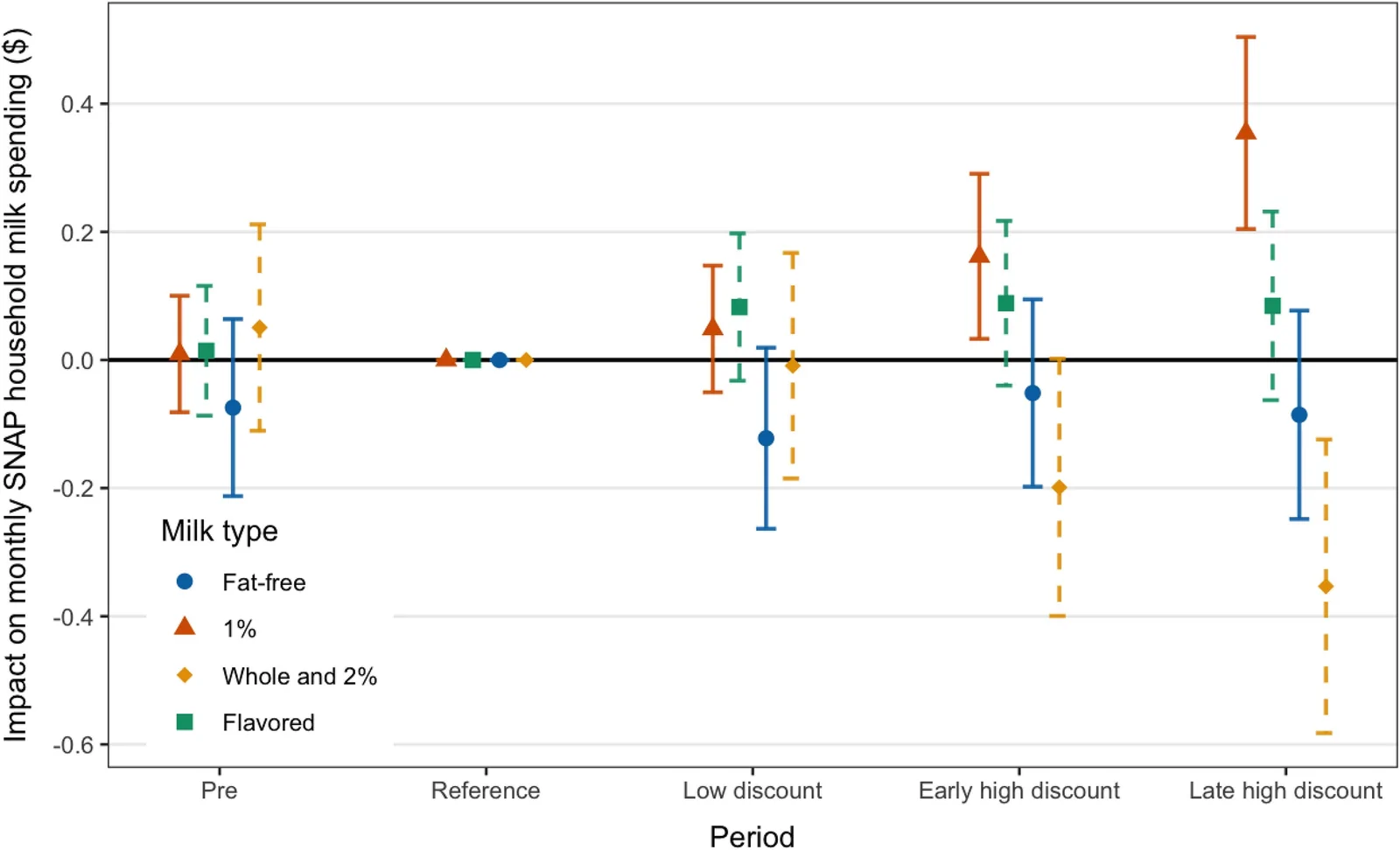
Impact of Add Milk on spending on milk types among SNAP households relative to non-SNAP households, 2023–2025. Coefficients and 95% CIs are shown from linear event study models comparing trends in spending on milk types between SNAP and non-SNAP households. December 2023, April 2024, and February 2025 are omitted from the sample as explained in the text. “Pre” indicates the pre-Add Milk launch period from May 2023 to October 2023 (6 months). “Low discount” indicates the 20% discount period from January 2024 to March 2024 (3 months). “Early high discount” indicates the first 7 months of the 40% discount period (May 2024 to November 2024). “Late high discount” indicates the next 7 months of the 40% discount period (December 2024 to July 2025, excluding February 2025. November 2023 is the reference period. Abbreviation: SNAP, Supplemental Nutrition Assistance Program.

Prior to the launch of Add Milk, changes in SNAP household spending on fat-free milk and 1% milk were not significantly different from changes in non-SNAP household spending (Figure 1). This provides evidence in support of the assumption that SNAP and non-SNAP households would have had similar trends in spending on milk types without the program. Full model results can be found in Table S1 (to access the Appendix, click on the Details tab of the article online).

After program launch, SNAP household spending on fat-free and 1% milk did not significantly change relative to non-SNAP household spending until the 40% discount was implemented in the early high discount period. By the late high discount period, SNAP household spending on 1% milk had increased from the reference period by $0.35 (95% CI, $0.20–$0.50) more than the increase in non-SNAP households. Prior to Add Milk, SNAP households spent an estimated $1.67 on 1% milk (Table 1), so this impact represents a 21.0% increase in spending on 1% milk. The increase in 1% milk spending happened concurrently with a decrease in whole and 2% milk spending. By the late high discount period, SNAP household spending on whole and 2% milk had decreased from the reference period by $0.35 (95% CI, −$0.58 to −$0.12) more than non-SNAP household spending on whole and 2% milk. Changes in spending on fat-free milk among SNAP households, however, were not significantly different from changes among non-SNAP households at any program period.

### Robustness analyses

We examined the influence of the sample restrictions by running the DID and event study models on households that always purchased $2 or more of milk at the retailer. Households that consistently purchase a large amount of milk at this retailer may depend less on other retailers, minimizing the potential bias from unobserved milk purchases elsewhere. Our results are similar when we restrict the sample to these households (Table S2 and Figure S1).

Additionally, we examined the robustness of our results to using the monthly number of units bought by a household as the outcome. This measure has limitations because each product size counts as 1 unit. Thus, for example, both 1 gallon of 1% milk and 1 half-gallon of 1% milk count as 1 unit. Similar to our results for spending on each milk type, the change in the number of 1% milk units purchased by SNAP households increased relative to the change for non-SNAP households, while the quantity of whole and 2% milk decreased (Table S3 and Figure S2).

We also examined whether our results changed when we did not control for total milk transactions. The DID and event study estimates from omitting this control are found in Table S4 and Figure S3; we found very similar estimates to our main results. Finally, we considered the robustness of our results to using indicators for each month instead of periods of multiple months. Table S5 and Figure S4 show that event study results based on month indicators are similar to the results from period indicators.

## Discussion

This study used information on transactions from a large retailer to evaluate the impact of a federally funded HFMI project (Add Milk) on the type of milk purchased by SNAP households. We found that, among households that regularly shop at this retailer, spending in SNAP households on targeted 1% milk increased by up to 21.0% more than changes in non-SNAP household spending, while spending on fat-free milk was not impacted. Spending on whole and 2% milk dropped concurrently with increases in 1% milk spending, but spending on flavored milk did not change as a result of the program. The program took over 6 months to have an impact on 1% milk spending and whole and 2% spending.

The impact of Add Milk on the value of spending for 1% milk (but not fat-free milk) is slightly higher than that of fruit and vegetable incentive programs. The USDA's Healthy Incentives Pilot (HIP) used a similar incentive amount ($0.30 for every SNAP dollar spent on targeted fruits and vegetables was credited back to the customer's EBT card) and reported that spending on targeted fruits and vegetables by households assigned to the incentive was 11% higher due to the program.^14^

One potential explanation for the slightly larger impacts of Add Milk than HIP is the program design. In contrast to Add Milk, which implemented incentives as an immediate discount, HIP incentives would have had to be used in a later transaction. The HIP incentives could have also been spent on any SNAP item, while the Add Milk discount only applied to qualifying milk. Another study that provided an immediate price discount on fruits and vegetables also found somewhat higher impacts than HIP (21% for fruits and 12% for vegetables).^15^

An additional explanation for the slightly lower impact of HIP is that customers have different own-price elasticities for 1% milk compared with fruits and vegetables. Even with an identical program design, a larger own-price elasticity for 1% milk than fruit and vegetables would result in a larger impact of incentives that reduce the effective price for the item. Consistent with this explanation, there is evidence that some milk products have larger own-price elasticities than healthier fruits and vegetables.^16^

Our finding that measurable changes in spending only emerged 6 months after the launch of the program may be due to a variety of factors. Since the Add Milk program at this retailer started by providing a 20% discount, we are unable to disentangle a slow increase in spending as a result of the program from a negligible impact of the smaller discount. After the switch to a 40% discount, however, target milk sales still took another 3 months to increase.

One explanation for this delayed program impact could be a lack of program awareness. Low and slowly increasing program awareness has also been found in fruit and vegetable incentive programs. Awareness among HIP participants increased from 62% partway through the first year of the program to 76% at the end of the first year.^5^ In the context of Rhode Island's Eat Well Be Well fruit and vegetable incentive program, only one-third of Rhode Island residents knew what the program did.^17^ Customer awareness of Add Milk may have come from in-store signs and observing discounts on receipts, but these signals might be overlooked the first few times a customer comes into contact with them. Additionally, awareness may have been spread over time by word-of-mouth. Information spread by word-of-mouth has been found in fruit and vegetable programs: Awareness of HIP increased over its first year among control households, likely through word-of-mouth spillover from treatment to control.^5^

Add Milk incentives for fat-free and 1% milk resulted in substitution away from whole and 2% milk, not away from flavored milk. Spending on flavored milk was substantially smaller overall than on whole and 2% milk, suggesting that it fills a different role in the SNAP household's diet. Many children receive flavored milk at school^18^; future work could examine spillover effects on flavored milk spending in contexts outside of food retailers.

## Conclusion

This study adds to what is known about incentivizing healthy eating in the United States by investigating a federally funded milk incentive program. Unique to incentive programs, it incentivizes foods beyond fruits and vegetables and distributes incentives as immediate point-of-sale discounts. We found significant increases in targeted lower-fat milk spending among SNAP households, corresponding decreases in higher-fat milk spending, and no impact on flavored milk spending.

## Supplementary Material

qxag193_Supplementary_Data

## Data Availability

Data for this research were obtained from a retailer under a Memorandum of Understanding, and per this agreement, cannot be made publicly available.
