# Supplementary material for: Impacts of incentivizing milk spending among SNAP participants
Source: Health Aff Sch. 2026 Jul 25;4(8):qxag193. doi: 10.1093/haschl/qxag193 (PMC13505516; doi:10.1093/haschl/qxag193)
Supplement: Supplementary file 1 [file qxag193_supplementary_data.zip › final_Appendix_07_08_26.docx]

**Appendix to “Impacts of Incentivizing Milk Spending among SNAP Participants”**

**Appendix Table S1.** Event study estimates of fat-free milk, 1% milk, whole and 2% milk, and flavored milk spending

|  | Dependent variables | | | | | | | |
| --- | --- | --- | --- | --- | --- | --- | --- | --- |
| Independent variables | Fat-free  milk ($) | | 1%  milk ($) | | Whole and 2%  milk ($) | | Flavored  milk ($) | |
|  | estimate | c.i. | estimate | c.i. | estimate | c.i. | estimate | c.i. |
|  |  |  |  |  |  |  |  |  |
| Pre | 0.04*** | (0.03, 0.05) | 0.00 | (-0.01, 0.01) | -0.06*** | (-0.08, -0.04) | 0.01* | (0.00, 0.03) |
| Low discount | 0.06*** | (0.05, 0.07) | -0.04*** | (-0.06, -0.02) | -0.05*** | (-0.07, -0.02) | 0.02*** | (0.01, 0.04) |
| Early high discount | 0.06*** | (0.04, 0.07) | -0.09*** | (-0.10, -0.07) | 0.00 | (-0.02, 0.03) | 0.02** | (0.01, 0.04) |
| Late high discount | 0.11*** | (0.09, 0.13) | -0.11*** | (-0.13, -0.10) | 0.07*** | (0.04, 0.10) | -0.07*** | (-0.08, -0.05) |
| Pre x SNAP | -0.07 | (-0.21, 0.06) | 0.01 | (-0.08, 0.10) | 0.05 | (-0.11, 0.21) | 0.01 | (-0.09, 0.12) |
| Low discount x SNAP | -0.12 | (-0.26, 0.02) | 0.05 | (-0.05, 0.15) | -0.01 | (-0.18, 0.17) | 0.08 | (-0.03, 0.20) |
| Early high discount x SNAP | -0.05 | (-0.19, 0.10) | 0.16* | (0.03, 0.29) | -0.20* | (-0.40, 0.00) | 0.09 | (-0.04, 0.22) |
| Late high discount x SNAP | -0.09 | (-0.25, 0.08) | 0.35*** | (0.20, 0.50) | -0.35** | (-0.58, -0.12) | 0.08 | (-0.06, 0.23) |
| Total milk spending | 0.13 | (0.11, 0.15) | 0.06* | (-0.01, 0.14) | 0.71*** | (0.65, 0.77) | 0.10*** | (0.08, 0.12) |
| Total milk transactions | -0.01 | (-0.08, 0.06) | 0.15 | (-0.13, 0.43) | -0.22* | (-0.43, -0.01) | 0.08* | (0.01, 0.15) |
| N | 3,929,952 |  | 3,929,952 |  | 3,929,952 |  | 3,929,952 |  |
| R2 | 0.13 |  | 0.07 |  | 0.69 |  | 0.12 |  |
| Number of households: | 163,748 |  |  |  |  |  |  |  |

* p<0.05, ** p<0.01, *** p<0.001

Note: estimates are relative to reference period (November 2023). “Pre” spans over May 2023- October 2023, “low discount” spans over January 2024 – March 2024, “early high discount” spans from May 2024- November 2024 and “late high discount” spans from December 2024 to July 2025 excluding February 2025.

**Appendix Table S2** Adjusted milk spending by SNAP and non-SNAP households, pre- and post-launch of the Add Milk program. Milk spending $2 or more each month. (Robustness check; SNAP 3,897 and non-SNAP 126,228)

|  | **SNAP** | | |  | **Non-SNAP** | | |  |
| --- | --- | --- | --- | --- | --- | --- | --- | --- |
|  | **Pre** | **Post** | **Difference** |  | **Pre** | **Post** | **Difference** | **DID (95% CI)** |
|  |  |  |  |  |  |  |  |  |
| 1% milk (number) | 1.89 | 2.03 | 0.14 |  | 1.97 | 1.87 | -0.10 | 0.23 (0.15, 0.32) |
| Fat-free milk (number) | 0.86 | 0.88 | 0.02 |  | 2.36 | 2.43 | 0.07 | -0.04 (-0.15, 0.05) |
| Whole and 2% (number) | 11.49 | 11.28 | -0.21 |  | 10.37 | 10.43 | 0.06 | -0.26 (-0.39, -0.13) |
| Flavored milk (number) | 1.93 | 1.98 | 0.05 |  | 1.45 | 1.48 | 0.03 | 0.07 (0.00, 0.14) |

Source: Authors’ analysis of household-month for households’ milk spending at participating Add Milk retailer. Notes: Averages and differences shown for households that used SNAP at the retailer every month (SNAP) and households that never used SNAP (non-SNAP) prior to and after launch of the Add Milk program. These households spent $2 or more each month on milk. Averages and differences adjusted for total milk spending and total milk transactions. Abbreviations: DID, difference-in-difference.

**Appendix Figure S1.** Impact of Add Milk on SNAP household spending on milk types relative to non-SNAP household spending, 2023-2025. Milk spending $2 or more each month. (Robustness check; SNAP 3,897 and non-SNAP 126,228)

**Appendix Figure S1**. Impact of Add Milk on SNAP household spending on milk types relative to non-SNAP household spending, 2023-2025. Households’ milk spending $2 or more each month. Coefficients and 95% confidence intervals are shown from linear differences-in-differences event study models comparing trends in spending on incentivized milk between SNAP and non-SNAP households December 2023, April 2024, and February 2025 are omitted from the sample as explained in the text. “Pre” indicates the pre-Add Milk launch period from May 2023 to October 2023 (6 months). “Low discount” indicates the 20% discount period from January 2024 to March 2024 (3 months). “Early high discount” indicates the first 7 months of the 40% discount period (May 2024 to November 2024). “Late high discount” indicates the next 7 months of the 40% discount period (December 2024 to July 2025 excluding February 2025. November 2023 is the reference period.

**Appendix Table S3.** Adjusted number of units of milk purchased by SNAP and non-SNAP households, pre- and post-launch of the Add Milk program

|  | **SNAP** | | |  | **Non-SNAP** | | |  |
| --- | --- | --- | --- | --- | --- | --- | --- | --- |
|  | **Pre** | **Post** | **Difference** |  | **Pre** | **Post** | **Difference** | **DID (95% CI)** |
|  |  |  |  |  |  |  |  |  |
| 1% milk (number) | 0.60 | 0.65 | 0.05 |  | 0.66 | 0.63 | -0.03 | 0.08 (0.05, 0.10) |
| Fat-free milk (number) | 0.19 | 0.20 | 0.01 |  | 0.68 | 0.67 | -0.01 | 0.00 (-0.01, 0.04) |
| Whole and 2% (number) | 3.64 | 3.56 | -0.08 |  | 3.18 | 3.14 | -0.04 | -0.04 (-0.09, -0.00) |
| Flavored milk (number) | 0.62 | 0.65 | 0.03 |  | 0.45 | 0.45 | 0.00 | 0.03 (0.00, 0.05) |

Source: Authors’ analysis of household-month for households’ number of milk purchased from participating Add Milk retailer. Notes: Averages and differences shown for households that used SNAP at the retailer every month (SNAP) and households that never used SNAP (non-SNAP) prior to and after launch of the Add Milk program. Averages and differences adjusted for total milk spending and total milk transactions. Abbreviations: DID, difference-in-difference.

**Appendix Figure S****2.** Impact of Add Milk on the number of units of different milk types purchased by SNAP households relative to non-SNAP households.

**Appendix Figure S2**. Impact of Add Milk on the number of units of different milk types purchased by SNAP households relative to non-SNAP households. Coefficients and 95% confidence intervals are shown from linear differences-in-differences event study models comparing trends in number of units bought of incentivized milk between SNAP and non-SNAP households. December 2023, April 2024, and February 2025 are omitted from the sample as explained in the text. “Pre” indicates the pre-Add Milk launch period from May 2023 to October 2023 (6 months). “Low discount” indicates the 20% discount period from January 2024 to March 2024 (3 months). “Early high discount” indicates the first 7 months of the 40% discount period (May 2024 to November 2024). “Late high discount” indicates the next 7 months of the 40% discount period (December 2024 to July 2025 excluding February 2025. November 2023 is the reference period.

**Appendix Table S4.** Adjusted milk spending by SNAP and non-SNAP households, pre- and post-launch of the Add Milk program. (model excludes the number of milk transactions as control variable)

|  | **SNAP** | | |  | **Non-SNAP** | | |  |
| --- | --- | --- | --- | --- | --- | --- | --- | --- |
|  | **Pre** | **Post** | **Difference** |  | **Pre** | **Post** | **Difference** | **DID (95% CI)** |
|  |  |  |  |  |  |  |  |  |
| 1% milk (number) | 2.11 | 2.23 | 0.12 |  | 1.96 | 1.87 | -0.09 | 0.21 (0.13, 0.29) |
| Fat-free milk (number) | 0.85 | 0.87 | 0.02 |  | 2.37 | 2.43 | 0.06 | -0.04 (-0.14, 0.05) |
| Whole and 2% (number) | 11.00 | 10.84 | -0.16 |  | 10.40 | 10.43 | 0.03 | -0.20 (-0.33, -0.07) |
| Flavored milk (number) | 2.23 | 2.25 | 0.02 |  | 1.46 | 1.45 | -0.01 | 0.03 (-0.03, 0.10) |

Source: Authors’ analysis of household-month for households’ milk spending at participating Add Milk retailer. Notes: Averages and differences shown for households that used SNAP at the retailer every month (SNAP) and households that never used SNAP (non-SNAP) prior to and after launch of the Add Milk program. Averages and differences adjusted for total milk spending and total milk transactions. Model excludes the number of milk transactions as control variable Abbreviations: DID, difference-in-difference.

**Appendix Figure S3.** Impact of Add Milk on spending on milk types among SNAP households relative to non-SNAP households, 2023-2025. (Robustness check: we estimated the model without the total number of milk transactions as a control variable)

**Appendix Figure S3**. Impact of Add Milk on spending on milk types among SNAP households relative to non-SNAP households, 2023-2025. We estimated the model without the total number of milk transactions as a control variable. Coefficients and 95% confidence intervals are shown from linear event study models comparing trends in spending on milk types between SNAP and non-SNAP households. December 2023, April 2024, and February 2025 are omitted from the sample as explained in the text. “Pre” indicates the pre-Add Milk launch period from May 2023 to October 2023 (6 months). “Low discount” indicates the 20% discount period from January 2024 to March 2024 (3 months). “Early high discount” indicates the first 7 months of the 40% discount period (May 2024 to November 2024). “Late high discount” indicates the next 7 months of the 40% discount period (December 2024 to July 2025 excluding February 2025. November 2023 is the reference period.

**Appendix Table S5:** Event study month estimates of fat-free, 1% milk, whole and 2% milk, and flavored milk spending

|  |  | Dependent Variables | | | | | | | | | | | | | |  |
| --- | --- | --- | --- | --- | --- | --- | --- | --- | --- | --- | --- | --- | --- | --- | --- | --- |
| Independent variables | Fat-free  Milk ($) | |  | 1%  milk ($) | | | |  | Whole and 2%  milk ($) | | |  | Flavored  milk ($) | | |  |
| Event time | estimate | c.i |  | estimate | | | c.i. |  | estimate | | c.i. |  | estimate | | c.i. |  |
| -7 | 0.06*** | (0.05, 0.08) |  | 0.04*** | | (0.02, 0.05) | |  | -0.10*** | | (-0.12, -0.07) |  | 0.00 | (-0.01, 0.02) | |  |
| -6 | 0.04*** | (0.03, 0.06) |  | 0.00 | | (-0.01, 0.02) | |  | -0.06*** | | (-0.09, -0.04) |  | 0.02* | (0.00, 0.03) | |  |
| -5 | 0.03*** | (0.01, 0.05) |  | -0.01 | | (-0.03, 0.01) | |  | -0.03* | | (-0.05, -0.00) |  | 0.00 | (-0.01, 0.02) | |  |
| -4 | 0.05*** | (0.04, 0.07) |  | -0.02* | | (-0.04, 0.00) | |  | -0.07*** | | (-0.09, -0.05) |  | 0.04*** | (0.02, 0.05) | |  |
| -3 | 0.02** | (0.01, 0.04) |  | -0.01* | | (-0.03, 0.00) | |  | -0.06*** | | (-0.09, -0.04) |  | 0.06*** | (0.04, 0.07) | |  |
| -2 | 0.04*** | (0.03, 0.06) |  | 0.03*** | | (0.01, 0.04) | |  | -0.03*** | | (-0.05, -0.01) |  | -0.03*** | (-0.05, -0.02) | |  |
| 1 | 0.07*** | (0.06, 0.09) |  | 0.00 | | (-0.02, 0.02) | |  | -0.05*** | | (-0.07, -0.02) |  | -0.03*** | (-0.04, -0.01) | |  |
| 2 | 0.06*** | (0.04, 0.07) |  | -0.06*** | | (-0.08, -0.04) | |  | -0.05*** | | (-0.08, -0.03) |  | 0.05*** | (0.04, 0.07) | |  |
| 3 | 0.05*** | (0.03, 0.07) |  | -0.06*** | | (-0.07, -0.05) | |  | -0.04** | | (-0.06, -0.02) |  | 0.05*** | (0.03, 0.06) | |  |
| 4 | 0.07*** | (0.05, 0.09) |  | -0.08*** | | (-0.11, -0.06) | |  | -0.03* | | (-0.06, -0.00) |  | 0.04*** | (0.03, 0.06) | |  |
| 5 | 0.05*** | (0.03, 0.06) |  | -0.10*** | | (-0.12, -0.08) | |  | 0.00 | | (-0.03, 0.02) |  | 0.06*** | (0.04, 0.07) | |  |
| 6 | 0.08*** | (0.06, 0.10) |  | -0.11*** | | (-0.12, -0.09) | |  | 0.02 | | (-0.00, 0.05) |  | 0.01 | (-0.01, 0.02) | |  |
| 7 | 0.07*** | (0.05, 0.09) |  | -0.07*** | | (-0.09, -0.06) | |  | -0.01 | | (-0.04, 0.01) |  | 0.02* | (0.00, 0.04) | |  |
| 8 | 0.03*** | (0.02, 0.05) |  | -0.08*** | | (-0.10, -0.06) | |  | 0.03. | | (-0.00, 0.06) |  | 0.02. | (-0.00, 0.03) | |  |
| 9 | 0.08*** | (0.06, 0.10) |  | -0.09*** | | (-0.11, -0.07) | |  | -0.02 | | (-0.05, 0.01) |  | 0.03*** | (0.01, 0.05) | |  |
| 10 | 0.04*** | (0.02, 0.06) |  | -0.07** | | (-0.12, -0.02) | |  | 0.05* | | (0.00, 0.09) |  | -0.01 | (-0.03, 0.01) | |  |
| 11 | 0.06*** | (0.04, 0.08) |  | -0.10*** | | (-0.13, -0.06) | |  | 0.09*** | | (0.05, 0.12) |  | -0.05*** | (-0.07, -0.03) | |  |
| 12 | 0.10*** | (0.08, 0.13) |  | -0.10*** | | (-0.12, -0.07) | |  | 0.05** | | (0.02, 0.08) |  | -0.06*** | (-0.08, -0.04) | |  |
| 13 | 0.13*** | (0.11, 0.15) |  | -0.14*** | | (-0.16, -0.11) | |  | 0.05* | | (0.01, 0.08) |  | -0.04*** | (-0.06, -0.02) | |  |
| 14 | 0.07*** | (0.05, 0.10) |  | -0.08*** | | (-0.11, -0.05) | |  | 0.08*** | | (0.04, 0.12) |  | -0.07*** | (-0.09, -0.05) | |  |
| 15 | 0.15*** | (0.12, 0.17) |  | -0.12*** | | (-0.13, -0.10) | |  | 0.02 | | (-0.01, 0.05) |  | -0.05*** | (-0.07, -0.03) | |  |
| 16 | 0.13*** | (0.10, 0.15) |  | -0.15*** | | (-0.18, -0.13) | |  | 0.04* | | (0.00, 0.07) |  | -0.01 | (-0.03, 0.01) | |  |
| 17 | 0.15*** | (0.12, 0.17) |  | -0.12*** | | (-0.14, -0.10) | |  | 0.17*** | | (0.14, 0.20) |  | -0.20*** | (-0.22, -0.18) | |  |
| -7 x SNAP | -0.13 | (-0.29, 0.03) |  | 0.07 | | (-0.05, 0.20) | |  | 0.03 | | (-0.19, 0.24) |  | 0.03 | (-0.12, 0.17) | |  |
| -6 x SNAP | -0.04 | (-0.20, 0.12) |  | -0.01 | | (-0.14, 0.11) | |  | 0.11 | | (-0.09, 0.32) |  | -0.06 | (-0.19, 0.08) | |  |
| -5 x SNAP | -0.09 | (-0.25, 0.07) |  | -0.02 | | (-0.14, 0.10) | |  | 0.18* | | (-0.02, 0.38) |  | -0.06 | (-0.20, 0.07) | |  |
| -4 x SNAP | -0.07 | (-0.22, 0.08) |  | -0.03 | | (-0.14, 0.08) | |  | 0.14 | | (-0.05, 0.33) |  | -0.04 | (-0.17, 0.09) | |  |
| -3 x SNAP | -0.03 | (-0.17, 0.12) |  | 0.00 | | (-0.10, 0.10) | |  | -0.14 | | (-0.32, 0.03) |  | 0.17** | (0.06, 0.29) | |  |
| -2 x SNAP | -0.09 | (-0.23, 0.06) |  | 0.05 | | (-0.05, 0.14) | |  | -0.01 | | (-0.18, 0.16) |  | 0.05 | (-0.06, 0.16) | |  |
| 1 x SNAP | -0.19* | (-0.34, -0.04) |  | 0.05 | | (-0.05, 0.16) | |  | 0.13 | | (-0.06, 0.32) |  | 0.00 | (-0.12, 0.13) | |  |
| 2 x SNAP | -0.06 | (-0.21, 0.09) |  | 0.05 | (-0.07, 0.16) | | |  | -0.01 | (-0.21, 0.18) | |  | 0.02 | | (-0.11, 0.15) | |
| 3 x SNAP | -0.12 | (-0.27, 0.03) |  | 0.04 | (-0.08, 0.16) | | |  | -0.14 | (-0.35, 0.06) | |  | 0.22** | | (0.07, 0.37) | |
| 4 x SNAP | 0.15* | (-0.01, 0.31) |  | 0.02 | (-0.12, 0.15) | | |  | -0.22* | (-0.43, -0.00) | |  | 0.05 | | (-0.09, 0.19) | |
| 5 x SNAP | 0.03 | (-0.13, 0.19) |  | 0.02 | (-0.12, 0.16) | | |  | -0.10 | (-0.32, 0.12) | |  | 0.04 | | (-0.10, 0.19) | |
| 6 x SNAP | -0.14* | (-0.30, 0.01) |  | 0.10 | (-0.04, 0.24) | | |  | -0.16 | (-0.38, 0.06) | |  | 0.20** | | (0.05, 0.35) | |
| 7 x SNAP | -0.15* | (-0.30, 0.01) |  | 0.13. | (-0.02, 0.27) | | |  | -0.09 | (-0.32, 0.14) | |  | 0.11 | | (-0.05, 0.26) | |
| 8 x SNAP | -0.07 | (-0.22, 0.09) |  | 0.21** | (0.05, 0.36) | | |  | -0.17 | (-0.40, 0.06) | |  | 0.03 | | (-0.12, 0.18) | |
| 9 x SNAP | -0.09 | (-0.25, 0.07) |  | 0.31*** | (0.15, 0.47) | | |  | -0.36** | (-0.60, -0.13) | |  | 0.15* | | (-0.01, 0.30) | |
| 10 x SNAP | -0.09 | (-0.26, 0.07) |  | 0.34*** | (0.18, 0.51) | | |  | -0.29* | (-0.54, -0.05) | |  | 0.04 | | (-0.12, 0.20) | |
| 11 x SNAP | -0.11 | (-0.29, 0.07) |  | 0.41*** | (0.25, 0.57) | | |  | -0.31* | (-0.56, -0.06) | |  | 0.01 | | (-0.15, 0.17) | |
| 12 x SNAP | -0.11 | (-0.28, 0.06) |  | 0.37*** | (0.20, 0.54) | | |  | -0.31* | (-0.56, -0.06) | |  | 0.05 | | (-0.11, 0.21) | |
| 13 x SNAP | -0.10 | (-0.28, 0.08) |  | 0.42*** | (0.24, 0.59) | | |  | -0.47*** | (-0.73, -0.21) | |  | 0.16. | | (-0.02, 0.33) | |
| 14 x SNAP | -0.04 | (-0.21, 0.14) |  | 0.36*** | (0.20, 0.53) | | |  | -0.38** | (-0.63, -0.12) | |  | 0.05 | | (-0.12, 0.22) | |
| 15 x SNAP | -0.12 | (-0.29, 0.06) |  | 0.29*** | (0.12, 0.46) | | |  | -0.35** | (-0.61, -0.09) | |  | 0.17* | | (0.01, 0.34) | |
| 16 x SNAP | -0.03 | (-0.20, 0.15) |  | 0.30*** | (0.13, 0.47) | | |  | -0.44*** | (-0.70, -0.19) | |  | 0.17* | | (-0.00, 0.34) | |
| 17 x SNAP | -0.10 | (-0.29, 0.09) |  | 0.33*** | (0.16, 0.50) | | |  | -0.22 | (-0.48, 0.05) | |  | -0.01 | | (-0.18, 0.16) | |
| Total milk spending | 0.13*** | (0.11, 0.15) |  | 0.06 | (-0.01, 0.14) | | |  | 0.71*** | (0.65, 0.77) | |  | 0.10*** | | (0.08, 0.12) | |
| Total milk transactions | -0.01 | (-0.08, 0.06) |  | 0.15 | (-0.13, 0.43) | | |  | -0.22* | (-0.43, -0.01) | |  | 0.08* | | (0.01, 0.15) | |
|  |  |  |  |  | | |  |  |  | |  |  |  | |  |  |
| N | 3,929,952 |  |  | 3,929,952 | | |  |  | 3,929,952 | |  |  | 3,929,952 | |  |  |
| Rsq | 0.13 |  |  | 0.07 | | |  |  | 0.69 | |  |  | 0.12 | |  |  |
| Number of households 163,748 | | | | | | | |  |  | |  |  |  | |  |  |

* p<0.05, ** p<0.01, *** p<0.001

Note: period “-1” is the reference period (November 2023). December 2023, April 2024, and February 2025 are omitted from the sample as explained in the text. Period 1 is the January 2024, period 4 is May 2024.

**Appendix Figure S4.** Impact of Add Milk on SNAP household spending on milk types relative to non-SNAP household spending, 2023-2025.

Note: Appendix Figure S4. Impact of Add Milk on SNAP household spending on milk types relative to non-SNAP household spending, 2023-2025. Coefficients and 95% confidence intervals are shown from differences-in-differences event study models comparing trends in spending on incentivized milk between SNAP and non-SNAP households. December 2023, April 2024, and February 2025 are omitted from the sample as explained in the text. The first dotted line shows the first full month of the program (January 2024) and the second dotted line shows the first full month after the program switched from 20% discount to 40% discount (May 2024).
